# PEDV and BVDV coinfection activates the NF-κB pathway by a TLR7-dependent mechanism

**DOI:** 10.3389/fmicb.2025.1684847

**Published:** 2025-10-27

**Authors:** Jinghua Cheng, Benqiang Li, Jie Tao, Ying Shi, Huili Liu

**Affiliations:** ^1^Institute of Animal Science and Veterinary Medicine, Shanghai Academy of Agricultural Science, Shanghai, China; ^2^Shanghai Key Laboratory of Agricultural Genetic Breeding, Shanghai, China; ^3^Shanghai Engineering Research Center of Pig Breeding, Shanghai, China

**Keywords:** coinfection, NF-κB pathway, Toll-like receptor, virus replication, immune response

## Abstract

**Introduction:**

In recent years, coinfections of porcine epidemic diarrhea virus (PEDV) with other enteric pathogens have been frequently observed in diarrheal piglets. However, the underlying mechanisms of pathogen–pathogen or pathogen–host interactions during such coinfections remain to be elucidated.

**Methods:**

In this study, an in vitro coinfection test of bovine viral diarrhea virus (BVDV) and PEDV was performed on model cells established before to explore the effect of coinfection on PEDV replication. Additionally, we used small interfering RNA (siRNA) assay to explore the role of TLRs related to NF-κB signaling in response to PEDV and BVDV coinfection.

**Results:**

We found that after preinfection with BVDV, PEDV replicates faster and more easily in PK15 cells. Further analysis revealed that coinfection of PEDV and BVDV significantly induced NF-κB activity, leading to increased translocation of the NF-κB p65 subunit from the cytoplasm to the nucleus. Moreover, TLR7 was identified as a key pattern recognition receptor in this process, mediating NF-κB activation and promoting the expression of inflammatory cytokines.

**Discussion:**

These results suggest that synergistic interactions between PEDV and BVDV during coinfection may strongly activate the host immune system and enhance virus replication. This study provides new insights into the immune modulation mechanisms underlying viral coinfections.

## 1 Introduction

Viral porcine diarrhea leads to diarrhea, vomiting, and dehydration in piglets and is the main cause of death in swine. It can be caused by a variety of porcine enteric pathogens, and porcine epidemic diarrhea virus (PEDV) is an important factor contributing to high mortality in piglets at home and abroad (Zhang et al., 2023). PEDV is a single-stranded RNA virus belonging to the family Coronaviridae in the order Nidovirales. Currently, coinfections with PEDV and other diarrhea-associated viruses are very common and may increase herd morbidity and mortality (Guo et al., 2024; Zhang et al., 2024). Our previous epidemiological surveys revealed that bovine viral diarrhea virus (BVDV), a bovine viral diarrhea virus, could be detected in PEDV-positive samples (Cheng et al., 2022). Our laboratory collected diarrhea samples from several pig farms in Shanghai between 2018 and 2023, and the detection rate of both PEDV and BVDV was 8.65%. BVDV has a broad host range and can infect various species such as cattle, camels, pigs and sheep (Ridpath, 2010). BVDV causes intestinal infections in pigs and has similar clinical symptoms as does PEDV, but its pathogenesis remains unclear (Tao et al., 2013). Upon entering the body, BVDV causes persistent infection and immune suppression, leading to invasion by other viruses.

During viral infection and replication, innate host immune responses serve as the first line of defense. A family of Toll-like receptors (TLRs) were the first discovered and major category of pattern-recognition receptors (PRRs) that sense foreign materials. In the TLR family, TLR1, TLR2, TLR4, TLR5, and TLR6 are located primarily on the cell surface plasma membrane and are responsible for recognizing bacterial products, such as lipids, lipoproteins and other microbial membrane components, whereas TLR3, TLR7, TLR8, and TLR9 are localized in the cell endosome and are involved mainly in the recognition of nucleic acids belonging to extracellular microorganisms (Aluri et al., 2021). TLRs utilize their common cytoplasmic Toll-interleukin-1 receptor (TIR) domain to transmit signals through the recruitment of downstream adaptors such as MyD88, TRIF, TRAM, and TIRAP. Some TLRs act as intermediates and initiate signaling cascades culminating in the activation of the transcription factor nuclear factor-kappa B (NF-κB), with consequent expression of an array of inflammatory cytokines (Meylan and Tschopp, 2006; Carmody and Chen, 2007). In the case of MyD88-dependent activation of NF-κB, MyD88 recruits IL-1 receptor-associated kinase (IRAK) to TLRs through interaction of the death domains, after which IRAK is activated, complexes with the E3 ubiquitin ligase TRAF6, and finally activates NF-κB (Takeda and Akira, 2004). Studies have shown that infection with either PEDV or BVDV alone can increase cytokine production through the NF-κB pathway (Cao et al., 2015; Fan et al., 2022). In our previous study, we demonstrated that PEDV and BVDV coinfection strongly activated the NF-κB signaling pathway, which induced higher production of inflammatory cytokines than did PEDV or BVDV infection alone (Cheng et al., 2022). However, whether some members of the TLR family mediate NF-κB activity response to PEDV and BVDV coinfection and the specific mechanism involved are not clear.

Here, an *in vitro* coinfection test of BVDV and PEDV was performed on model cells established before to explore the effect of coinfection on PEDV replication. We investigated the role of TLRs related to NF-κB signaling in response to PEDV and BVDV coinfection. We demonstrated the involvement of a TLR7-dependent signaling pathway in NF-κB activation upon PEDV and BVDV coinfection, which promotes the expression of inflammatory cytokines and may promote PEDV replication. These findings provide a new mechanistic explanation for the immune pathogenic mechanism of diarrhea virus coinfection leading to disease severity.

## 2 Materials and methods

### 2.1 Cells, viruses, reagents and antibodies

PEDV strain JS-2/2014, BVDV-2 strain SH-28 and PK15 cells were prepared as previously described (Cheng et al., 2022). The cells were grown in DMEM (Gibco, Grand Island, NY, United States) supplemented with 10% fetal bovine serum (FBS) (Gibco) at 37 °C and 5% CO_2_. Hydroxychloroquine (HCQ) sulfate, a small molecule inhibitor of TLR7 was purchased from Selleck (Houston, TX, United States) (Lamphier et al., 2014). A Toll-like Receptor Antibody Kit (48697), and antibodies against p65 (8242), Myd88 (4283), IRAK4 (4363), TRAF6 (8028), and β-actin (3700) were purchased from Cell Signaling Technology (Danvers, MA, United States). The polyclonal antibody against BVDV E2 protein was purchased from Bioss Biotechnology (Beijing, China). The monoclonal antibody against the PEDV N protein was purchased from BioNote (Hwaseong-si, South Korea).

### 2.2 Viral titration, quantitative real-time PCR and ELISA

To evaluate whether PEDV growth is affected by BVDV coinfection, PK15 cells were seeded in 35-mm dishes overnight and infected with PEDV (1 MOI) or inoculated with BVDV (1 or 0.01 MOI) for 6 h and then infected with PEDV. After 1 h, the medium was replaced with fresh DMEM containing 2 μg/mL trypsin (Promega, Madison, WI, United States). The supernatant of the infected culture was harvested at 12, 24, 36, and 48 h postinfection (hpi) for TCID_50_ determination. Viral genome copies of PEDV were quantified with a 7500 real-time PCR system (ABI, Madison, United States). Total RNA was extracted from the cells using TRIzol Reagent (Thermo Fisher Scientific, Waltham, MA, United States) and reverse transcribed into cDNA using a SuperScript III kit (Thermo Fisher Scientific). Quantitative real-time PCR (qRT-PCR) was carried out using SYBR Premix Ex Taq II (TaKaRa Bio, Shiga, Japan) with the specific primer pairs. The sequences of primers used for PEDV N gene and TLRs amplification were listed in Table 1, and primers for cytokines amplification were previously describe. The relative expression levels were determined and normalized to the reference gene β-actin levels by using the 2^−ΔΔCT^ method (Schmittgen and Livak, 2008). The protein levels of IL-6, IL-8, IL-18, and TNF-α in cell culture supernatants were determined using porcine ELISA kits (FineTest, Wuhan, China) according to the manufacturer’s protocol.

**Table 1:** Primers used in this study.

Name	Primer nucleotide sequence (5′→3′)
TLR3-F	GGAAATCTGTTTGGCCTTATACTGA
TLR3-R	TAAAACTGTGAGGTTTGTTCCTTGC
TLR7-F	TAAAATTGCTGACCTAAGGGTGTTC
TLR7-R	CTTGCAACTTCGACCATATTCATC
TLR8-F	ATGTTCCTTCAGTCGTCAATGC
TLR8-R	TTGCTGCACTCTGCAATAACT
TLR9-F	CTGCCTTCCTACCCTGTGAG
TLR9-R	GGATGCGGTTGGAGGACAA
N-F	ATGCTTCTTCTCTGCTGACG
N-R	TCTTCTCTGCTGACGCTTCT
β-actin-F	TGGGTCAGAAGGACTCCTATG
β-actin-R	CAGGCAGCTCATAGCTCTTCT

### 2.3 Immunofluorescence assays

PK15 cells were seeded into 24-well plates overnight and infected with PEDV or coinfected with BVDV. p65 nuclear translocation was detected via indirect immunofluorescence assay (IFA). Briefly, cells were fixed with 4% formaldehyde for 30 min at room temperature, permeabilized with 0.1% Triton X-100 for 10 min, and incubated in blocking buffer. Cells were then incubated at 37 °C with a rabbit anti-p65 monoclonal antibody (1:1,000) for 1 h and subsequently incubated with a FITC-conjugated goat anti-rabbit IgG (1:200) for 30 min. The cell nuclei were stained with 4′,6′-diamidino-2-phenylindole (DAPI) (Beyotime Biotechnology, Shanghai, China). The fluorescence signals were visualized using a fluorescence microscope (Carl Zeiss, Oberkochen, Germany).

### 2.4 RNA interference

Small interfering RNA (siRNA) molecules targeting TLR7 were used to knock down endogenous TLR7 expression. (siTLR7, 5′-GCCCAUUGAAACCAAGAAAUU-3′; nontargeting control siRNA, 5′-UUCUCCGAACGUGUCACGUTT-3′). PK15 cells grown to 40–50% confluence in 6-well plates were transfected with 100 nmol of siRNA using 3 μL of Lipofectamine 3000 (Thermo Fisher Scientific) in OptiMEM (Gibco). After 48 h of transfection, the cells were infected with PEDV or coinfected with BVDV at an MOI of 1 for 24 h and then harvested for analysis by Western blot or IFA. The siRNAs were obtained from Gene Pharma Co. (Shanghai, China).

### 2.5 Western blot assay

Western blot was used to investigate TLR pathway activation upon PEDV or BVDV infection. Six-well plates were seeded with PK15 cells overnight and infected with PEDV or BVDV or coinfected with both at an MOI of 1. Uninfected cells served as the mock-infected group. The cells were harvested at the indicated time points and lysed in RIPA buffer (Beyotime). The cell lysates were separated by SDS-PAGE, transferred to nitrocellulose filter membranes (Millipore, Billerica, MA) and incubated with primary antibodies, including rabbit monoclonal anti-TLRs (TLR3/7/8/9), rabbit monoclonal anti-Myd88, rabbit monoclonal anti-IRAK4, rabbit monoclonal anti-TRAF6, and rabbit monoclonal anti-β-actin overnight at 4 °C, followed by incubation with an HRP-conjugated goat anti-rabbit IgG antibody at room temperature for 1 h. The protein bands were detected using enhanced chemiluminescence detection kits (Thermo Fisher Scientific), visualized using an Amersham Imager 600 (Cytiva Sweden AB, United States) and quantified with ImageJ software (NIH, Bethesda, MD, United States).

### 2.6 Drug treatment

Drug experiments were conducted to inhibit the TLR7 pathway. PK15 cells were treated with or without the drug HCQ at 1 μM, and coinfected with PEDV and BVDV at an MOI of 1. At 1 h of virus infection, the cells were washed, and the medium was replaced with maintenance medium containing 1 μM HCQ. The supernatants were subsequently harvested for TCID_50_ assay, and the cells were subjected to Western blot and qPCR analyses at the indicated time points.

### 2.7 Statistical analysis

GraphPad Prism 6 software (GraphPad Software Inc., San Diego, CA, United States) was used for the data analyses and graph creation. All the data are presented as the means ± standard deviations (SD). The significance was determined with either an unpaired two-tailed independent Student’s t test for comparisons between two groups or one-way ANOVA for comparisons among multiple groups. *p* < 0.05 was considered to be statistically significant.

## 3 Results

### 3.1 Coinfection of PEDV with BVDV facilitates the proliferation of PEDV

**Figure 1:**
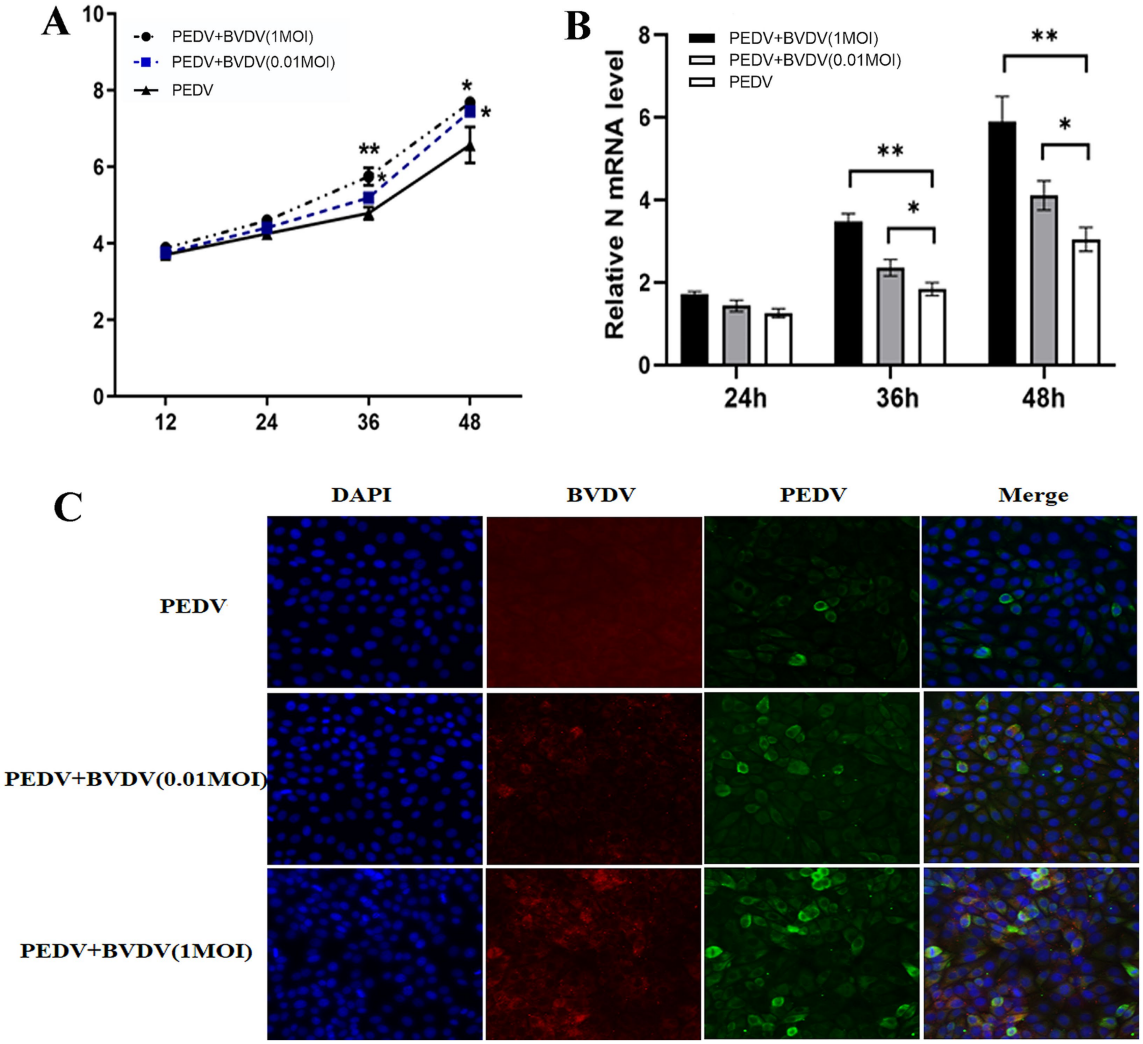
BVDV infection facilitates the proliferation of PEDV. PK15 cells were monoinfected with PEDV, or inoculated with BVDV for 6 h and then infected with PEDV. **(A)** At 12, 24, 36, and 48 hpi, PEDV virus titers were determined using a TCID_50_ assay. **(B)** PEDV N mRNA levels were detected by qPCR at 24, 36, and 48 hpi. **(C)** At 24 hpi, cells were fixed and stained for IFA. Green, PEDV N protein; red, BVDV E2 protein; blue, DAPI-stained for the nucleus. The results are representative of three independent experiments (^*^p < 0.05 and ^**^p < 0.01).

We use PK15 model cells to detect the impact on PEDV replication, as PK15 cells can be infected by PEDV and BVDV simultaneously and effectively. Because BVDV infection can cause persistent infection that may affect the production of other viruses, the cells were inoculated with BVDV for 6 h and then infected with PEDV, the PEDV monoinfected group was used as the control. TCID_50_ and qRT-PCR were used to determine the proliferation of PEDV. As shown in Figure 1A, after preinfection with BVDV, the titers of PEDV were higher at 36 and 48 hpi, and the increase was more obvious at high MOI of BVDV infection. Specifically, the viral titer of PEDV was increased 1.3-fold (0.01 MOI) and 8.7-fold (1 MOI) at 48 hpi (*p* < 0.05). Similar qPCR results also revealed that the mRNA level of the PEDV N gene was increased by 35% (0.01 MOI) and 93% (1MOI) at 48 hpi (Figure 1B). Moreover, IFA was performed to confirm the coinfection and the proliferation of PEDV. At 24 hpi, with high-MOI BVDV coinfection, PEDV exhibited intense immunofluorescence signals in cells (Figure 1C). In conclusion, these results indicate that after preinfection with BVDV, PEDV replicates faster and more easily in PK15 cells, and the ability of high-MOI BVDV to facilitate PEDV replication was greater than that of low-MOI BVDV.

### 3.2 Coinfection of PEDV with BVDV enhances nuclear translocation of p65

**Figure 2:**
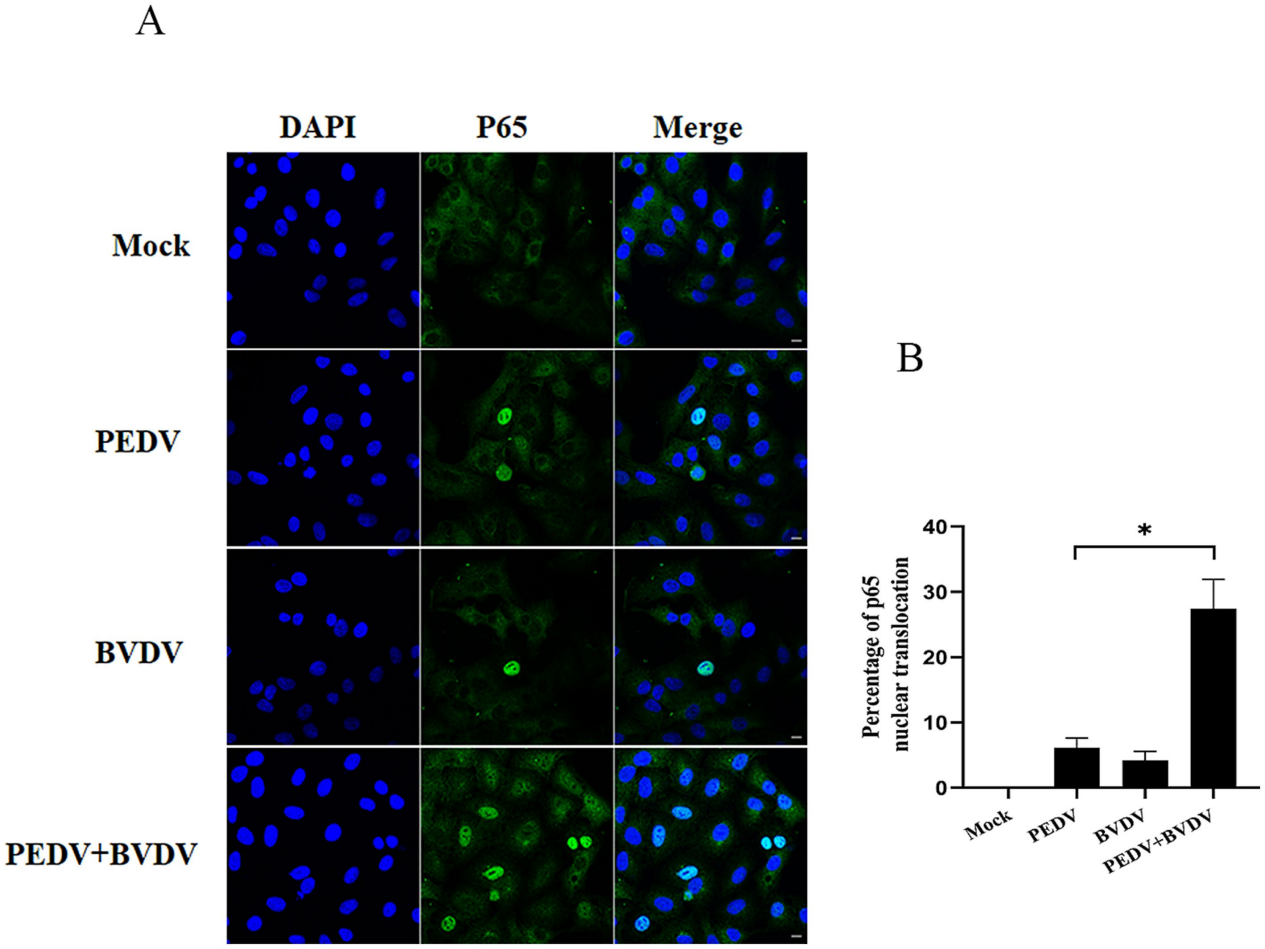
Coinfection of PEDV with BVDV enhances p65 translocation. PK15 cells were monoinfected with PEDV, or BVDV or coinfected with both at an MOI of 1 for 24 h, and p65 nuclear translocation was visualized by a converted fluorescence microscope. **(A)** p65 was stained with FITC-labeled goat anti-rabbit IgG (green), and nuclei were stained with DAPI (blue) (scale bar = 10 μm). **(B)** Percentage of p65 nuclear translocation. The results are representative of three independent experiments (^*^p < 0.05 and ^**^p < 0.01).

To confirm that PEDV coinfection with BVDV could enhance NF-κB activation, we studied the effect of PEDV monoinfection or coinfection with BVDV on the nuclear translocation of the NF-κB subunit p65 by confocal microscopy. As shown in Figure 2, in uninfected cells, p65 was expressed in the cytoplasm. Upon PEDV monoinfection or coinfection with BVDV at 24 hpi, p65 puncta were successfully detected in the nucleus. Moreover, compared to PEDV monoinfection, PEDV and BVDV coinfection caused increased p65 translocation from the cytoplasm to the nucleus, with the percentage rising from 6.13 to 27.46%. These results illustrate that compared with PEDV monoinfection alone, coinfection with PEDV and BVDV can strongly enhance NF-κB activation.

### 3.3 PEDV and BVDV coinfection upregulate TLR7 in cells

**Figure 3:**
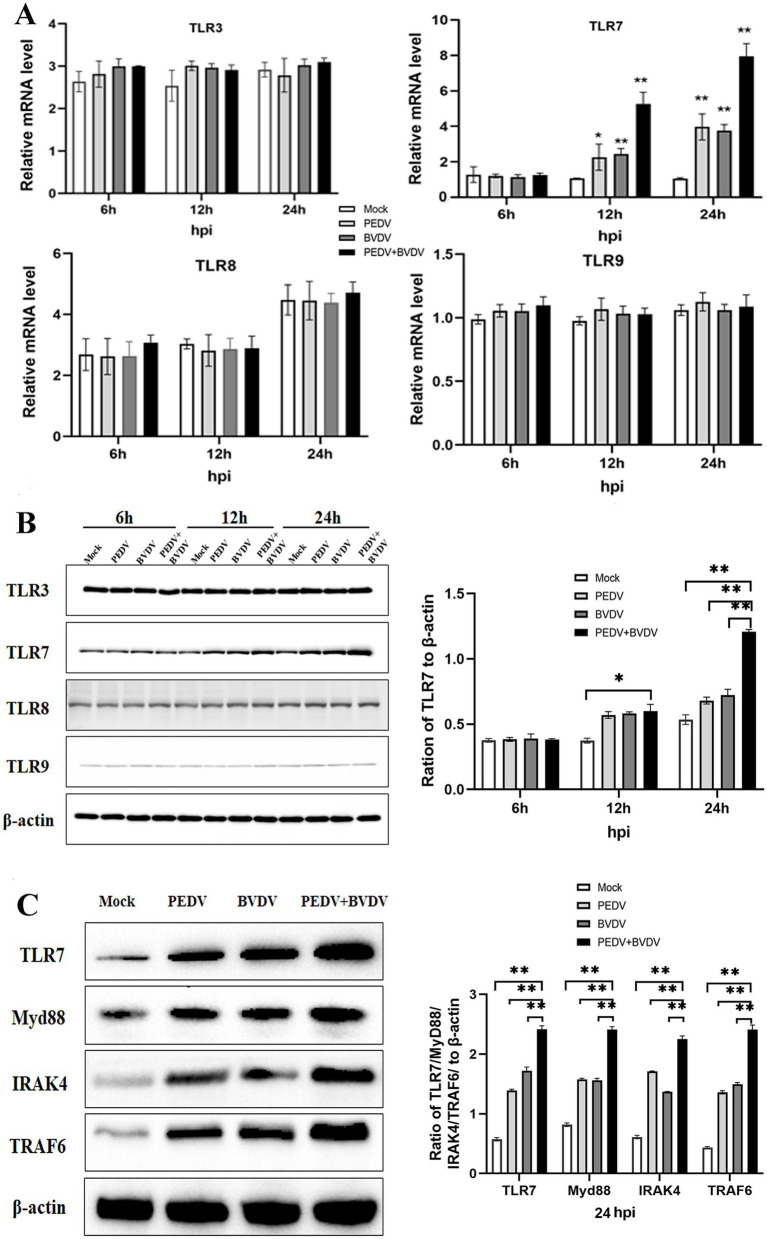
PEDV and BVDV coinfection upregulates TLR7 expression. PK15 cells were monoinfected with PEDV, or coinfected with BVDV at an MOI of 1 for 6, 12, 24 h. **(A,B)** TLR family members expression was detected via qPCR **(A)** and Western blotting **(B)** during the time course of virus infection. **(C)** TRL7, Myd88, IRAK4, and TRAF6 expression was analyzed via Western blotting at 24 hpi (^*^p < 0.05 and ^**^p < 0.01).

Endosomal TLR signaling, mainly regarding TLR3, TLR7, TLR8, and TLR9, plays a role in the activation of NF-κB, which controls an array of pro-inflammatory genes transcription and translation. Thus, the expression of TLRs was observed at 6, 12, 24 hpi. In the PEDV group and the coinfection group, the mRNA level of TLR7 was upregulated and peaked at 24 hpi, whereas no obvious changes were observed in the expression of other TLRs (Figure 3A). This trend was similar to that observed via the Western blot analysis. PEDV and BVDV coinfection rise up TLR7 expression the most significantly; however, TLR3 and TLR8 expression did not differ between the virus infection group and the control group, and TLR9 was expressed at relatively low levels in these cells (Figure 3B). As MyD88 associates with the TIR domain of TLR7, and recruits IRAK and TRAF6 to form complexes, we measured the changes in MyD88, and downstream signaling molecules IRAK4 and TRAF6 with PEDV/BVDV coinfection. As shown in Figure 3C, the expression of MyD88, TRAF6, and IRAK4 was obviously elevated by PEDV and BVDV coinfection at 24 hpi compared with that in control cells, while PEDV monoinfection slightly upregulated the expression of these downstream molecules. These results indicate that the TLR7/MyD88 pathway is relevant to PEDV and BVDV coinfection.

### 3.4 TLR7-MyD88 signaling mediates PEDV/BVDV coinfection-induced NF-κB activation

**Figure 4:**
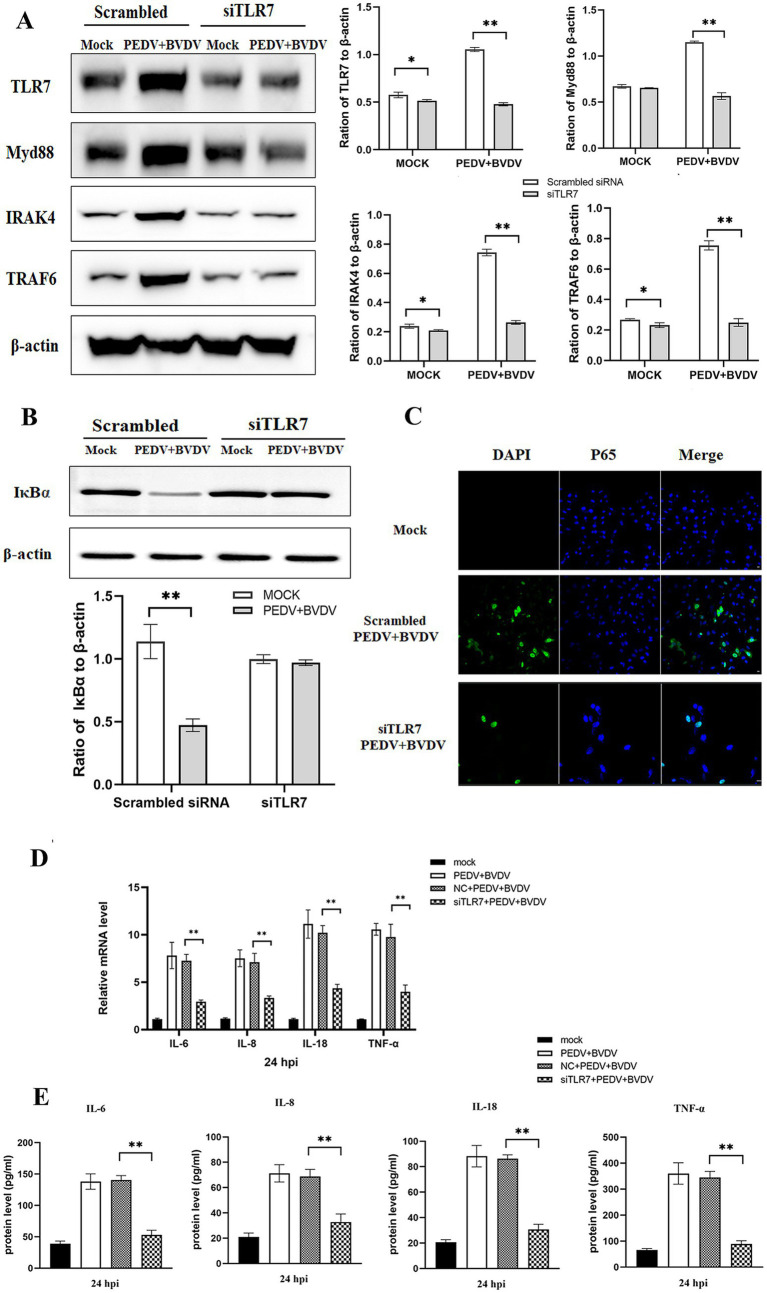
PEDV and BVDV coinfection induces NF-κB activation through the TLR7/Myd88 pathway. PK-15 cells were transfected with scrambled siRNA or siRNA specific for TLR7 (100 nmoL/mL) for 48 h and then coinfected with PEDV and BVDV for 24 h. **(A)** TRL7, Myd88, IRAK4, and TRAF6 expression was analyzed via Western blotting. **(B,C)** IκBα degradation and NF-κB p65 translocation were detected by Western blotting **(B)** and IFA **(C)**. **(D)** The relative expression levels of IL-6, IL-8, IL-18, and TNF-α were quantified by qPCR. **(E)** The cell supernatants were collected for detection of IL-6, IL-8, IL-18, and TNF-α using ELISA. The results are representative of three independent experiments (^*^p < 0.05 and ^**^p < 0.01).

To evaluate whether PEDV/BVDV coinfection activated the NF-κB pathway through TLR7 signaling, PK-15 cells were transfected with siTLR7 for 48 h and then infected with PEDV/BVDV for 24 h. We found that blockade of TLR7 reduced the expression of TLR7, Myd88, IRAK4, and TRAF6 (Figure 4A). We also measured IκBα levels in the cytoplasmic extracts and the degree of nuclear translocation of NF-κB p65. The results revealed that the degradation of IκBα was inhibited in siTRL7 -transfected cells following PEDV/BVDV coinfection, and that the nuclear translocation of P65 was reduced in siTRL7-transfected cells (Figures 4B,C). Our previous study demonstrated that NF-κB is pivotal in the induction of high expression of inflammatory cytokines by PEDV and BVDV coinfection (Cheng et al., 2022). Therefore, we measured the mRNA and protein levels of IL-6, IL-8, IL-18, and TNF-α in the presence of TLR7 siRNA transfection following PEDV and BVDV coinfection. The inflammatory cytokine expression at protein level showed similar tendencies as in the mRNA levels (Figures 4D,E). The results showed that TLR7 knockdown restricted the expression of IL-6, IL-8, IL-18, and TNF-α production, compared to the nontargeting siRNA transfection group at 24 hpi. These data indicate that TLR7 signaling mediates PEDV/BVDV coinfection-induced NF-κB activation, which in turn upregulated cytokine expression intensely.

### 3.5 PEDV/BVDV coinfection-induced TLR7 activation is closely related to viral replication

**Figure 5:**
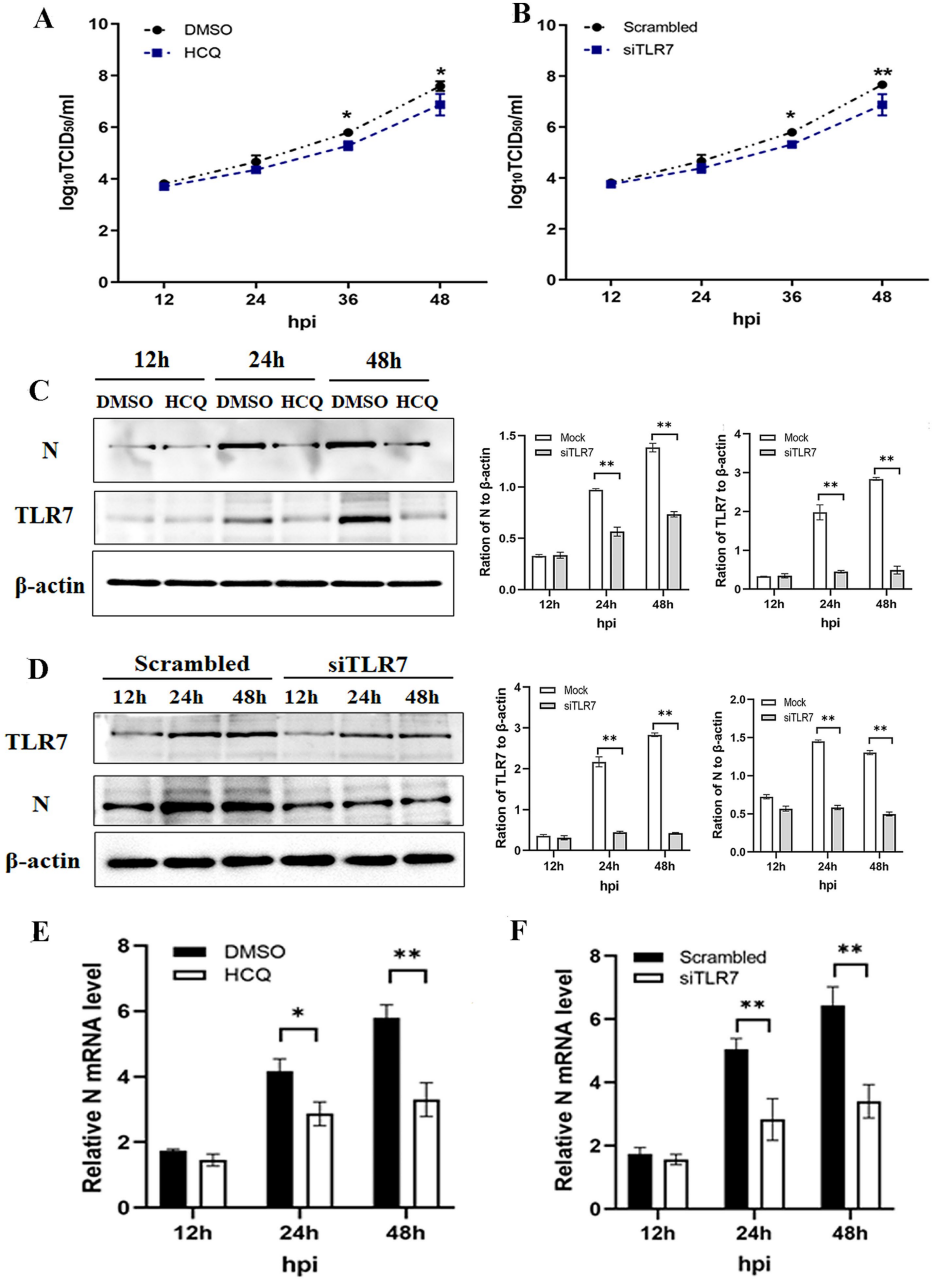
PEDV/BVDV coinfection-induced TLR7 activation is closely related to viral replication. PK-15 cells were pretreated with or without the TLR7 inhibitor HCQ for 2 h, and then coinfected with PEDV and BVDV for 24 hpi. **(A)** PEDV titers and **(C)** N protein expression and **(E)** mRNA level of N protein was determined using a TCID_50_ assay, Western blotting and qPCR, respectively. PK-15 cells were transfected with siTLR7 for 48 h, and then coinfected with PEDV and BVDV for 24 hpi. **(B)** PEDV titers and **(D)** N protein expression and **(F)** mRNA level of N protein was determined using a TCID_50_ assay, Western blotting and qPCR, respectively. The results are representative of three independent experiments (^*^p < 0.05 and ^**^p < 0.01).

The enhanced inflammatory activity is thought to contribute to disease severity. To investigate the effect of the TLR7-mediated inflammatory response on PEDV infectivity or virus progeny production, PK-15 cells were pretreated with or without the TLR7 inhibitor for 2 h and then infected with PEDV and BVDV. As shown in Figure 5A, PEDV grew similarly in cells with or without HCQ pretreatment, but the PEDV titer in presence of the HCQ was significantly lower than that in the absence of HCQ pretreatment at 36 and 48 hpi. Consistently, Western blotting and qRT-PCR analysis of the PEDV N protein confirmed the inhibitory effect of HCQ on PEDV replication, as it was significantly reduced at 24 and 48 hpi (Figures 5C,E). To exclude the HCQ itself impacting viral replication, we repeated the experiments in TLR7-knockdown cells. As shown in Figures 5B,D,F, the suppression of PEDV replication could also be found in siTLR7-transfected cells. These data confirmed that TLR7 inhibition exerts a suppressive effect on both viral replication and protein synthesis.

## 4 Discussion

Porcine diarrhea can be caused by many pathogens, and viral coinfections occur frequently in the clinical setting. Various coinfection patterns have been observed, and the most frequent coinfections are PEDV/PDCoV, PEDV/PKV, and PEDV/PoRV, which have been reported previously (Zhao et al., 2016; Zhang et al., 2024). Coinfections with multiple viruses may have more severe consequences than single-virus infections do. For example, porcine parvovirus (PPV) infection has been shown to provide a better *in vivo* environment for PCV2 infection, and coinfection with PCV2 and PPV may stimulate cytokine production and provide target cells for PCV2 replication (Kim et al., 2006). Similarly, PCV2 is easily detectable in pigs infected with PEDV, and PCV2 may enhance PEDV-induced disease and lesions, as more severe clinical symptoms occur in coinfection situations (Jung et al., 2006). In this study, we preinfected cells with BVDV for 6 h and then infected with PEDV. We found the synergistic effects, as BVDV could enhance the infection and replication of PEDV.

TLRs are recognized as significant contributors to inflammation caused by pathogens. During rabies virus infection, the expressed heat shock proteins (HSPs) can use TLR2/4 to activate the Toll/IL-1 receptor signaling pathway to participate in antiviral response (Vabulas et al., 2001). The Flaviviridae family of positive-sense RNA viruses, such as Zika virus (ZIKV), dengue virus (DENV), and West Nile virus (WNV), can be sensed by TLR3/7/8, and their agonists have been used to control virus replication and attenuate viral symptoms (Tsai et al., 2009; Suthar et al., 2013; Vanwalscappel et al., 2018). While different TLRs are involved in triggering innate antiviral immunity for viral clearance, they have also been linked to cytokine storms and facilitate virus replication. The cytokine surge that TLRs induce occurs mainly through the activation of TLR3, TLR4, TLR7, and TLR8 receptors (Stegeman et al., 2025). For example, compared with infection with low pathogenicity influenza virus (IAV) strains, lethal IAV strain infection preferentially upregulated TLR3 to a greater extent, which induced dysregulation of cytokines such as IL-6 and TNF-α and enhanced viral replication (Kalaiyarasu et al., 2016; Huo et al., 2018). During SARS-COV-2 infection, TLR7/8 recognizes the ssRNA, and when it replicates to dsRNA, it is recognized by TLR3. Activation of such TLRs has been shown to induce the cytokine storm and bring to systemic circulation, which can cause multiple organ failure, leading to disease severity and mortality (Manik and Singh, 2022). Both PEDV and BVDV have ssRNA genomes, and TLR7 recognizes ssRNA, subsequently activating an immune response. Our results revealed that coinfection with the two diarrhea viruses activated the TLR7-Myd88 signaling pathway, including increasing the expression of Myd88, IRAK4, and TRAF6 in PK-15 cells. In addition, blockade of TLR7 restricted the expression of IL-6, IL-8, IL-18, and TNF-α production, showing a positive correlation between the inflammatory agents of the immune response and TRL7-Myd88 signaling.

NF-κB is a branch of the signaling pathway downstream of TLRs. Numerous viruses, including rabies virus, nephropathogenic infectious bronchitis virus (NIBV), herpes simplex virus type 1 (HSV-1) and SARS-CoV-2 can induce NF-κB activation by the TLR-mediated pathway (Takeda et al., 2011; Li et al., 2022; Xie et al., 2024). In unstimulated cells, NF-κB dimmers (mostly p65/p50 dimers) are localized in the cytoplasm as a complex with the IκB proteins. Upon stimulation, IκB is ubiquitinated and subsequently degraded, thus, the released NF-κB subunits translocate to the nucleus and induce the transcription of target genes. Here, we found that PEDV with BVDV coinfection caused more p65 translocation from cytoplasm to nucleus, consistent with our prior study indicating stronger activation of the NF-κB signaling under coinfection conditions. Furthermore, in cells transfected with siTLR7, both the degradation of IκBα and the nuclear translocation of p65 induced by PEDV and BVDV coinfection were suppressed. These results suggest that the activation of the NF-κB pathway during PEDV and BVDV coinfection is dependent on TLR7 signaling.

In summary, the aim of this study was to explore the potential mechanism whereby coinfection induce intensive NF-κB activation in cells. We extended our previous findings and demonstrated PEDV and BVDV coinfection stimulates NF-κB activity via the TLR7/Myd88 pathways, meanwhile, PEDV/BVDV coinfection-induced TLR7 activation can facilitate virus replication. Understanding the TLR-dependent effects on NF-κB activation should provide information regarding the molecular pathogenesis of virus coinfection.

## Data Availability

The original contributions presented in the study are included in the article/Supplementary material, further inquiries can be directed to the corresponding author.
