# Supplementary figures and images for: PEDV and BVDV coinfection activates the NF-κB pathway by a TLR7-dependent mechanism
Source: Front Microbiol. 2025 Oct 27;16:1684847. doi: 10.3389/fmicb.2025.1684847 (PMC12597931; doi:10.3389/fmicb.2025.1684847)

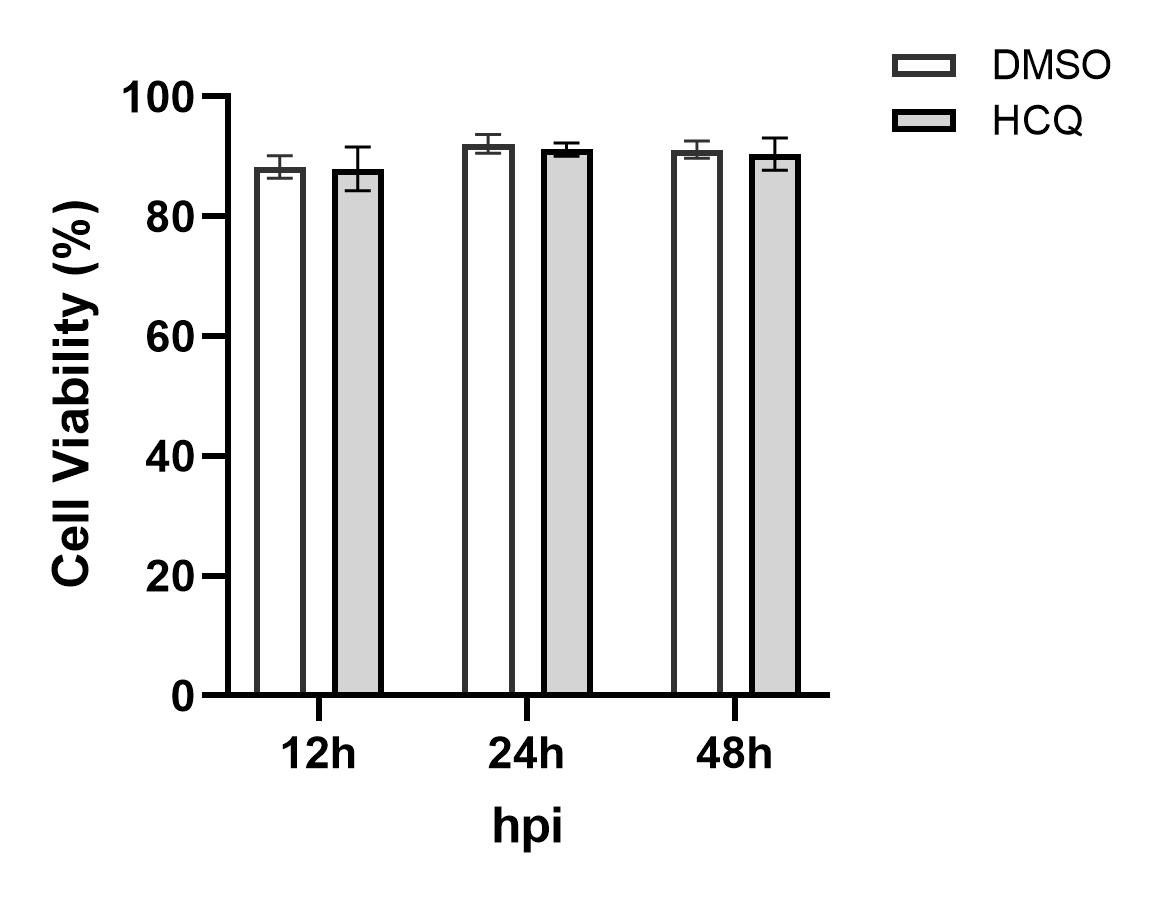

Supplement: Supplementary file 1 [file Image_1.TIF]
